# Circ_100565 promotes proliferation, migration and invasion in non-small cell lung cancer through upregulating HMGA2 via sponging miR-506-3p

**DOI:** 10.1186/s12935-020-01241-8

**Published:** 2020-05-12

**Authors:** Li Li, Haitao Wei, Haifeng Zhang, Feng Xu, Guowei Che

**Affiliations:** 1 Department of Thoracic Surgery, West China Hospital, Sichuan University, No. 37 Guoxue Lane, Wuhou District, Chengdu, 610041 Sichuan China; 2 School of Nursing and Health, Henan University, Kaifeng, 475001 Henan China; 3 Department of Thoracic Surgery, Huaihe Hospital of Henan University, Kaifeng, 475000 Henan China; 4 Department of Respiratory, Huaihe Hospital of Henan University, Kaifeng, 475000 Henan China

**Keywords:** NSCLC, circ_100565, miR-506-3p, HMGA2, Progression

## Abstract

**Background:**

Circular RNAs (circRNAs) play a vital role in the development of various cancers. Circ_100565 was found to be a highly expressed circRNA in non-small cell lung cancer (NSCLC) tissues screened by microarray profiles of circRNAs. However, the role of circ_100565 in NSCLC still remains unknown.

**Methods:**

Microarray analysis was used to screen for differentially expressed circRNAs in NSCLC tissues. The expression levels of circ_100565, microRNA-506-3p (miR-506-3p) and high mobility group AT-hook 2 (HMGA2) were measured by quantitative real-time polymerase chain reaction (qRT-PCR). Cell proliferation was detected by cell counting kit-8 (CCK-8) and colony formation assays. Transwell assay was used to determine the migration and invasion of cells. Besides, Western blot (WB) analysis was performed to assess the levels of proliferation and metastasis-related proteins and HMGA2 protein. Moreover, animal experiments were used to confirm the effect of circ_100565 on NSCLC tumor growth in vivo. In addition, the interaction between miR-506-3p and circ_100565 or HMGA2 was confirmed by dual-luciferase reporter, RNA immunoprecipitation (RIP) assay or biotin-labeled pull-down assay.

**Results:**

Circ_100565 was upregulated in NSCLC, and its high expression was positively associated with the poor overall survival of NSCLC patients. Silencing of circ_100565 suppressed the proliferation, migration and invasion of NSCLC cells in vitro and reduced the tumor growth of NSCLC in vivo. Circ_100565 could sponge miR-506-3p, and miR-506-3p could target HMGA2. Moreover, miR-506-3p inhibitor or HMGA2 overexpression could reverse the inhibition effect of circ_100565 knockdown on NSCLC progression.

**Conclusion:**

Circ_100565 increased HMGA2 expression to promote proliferation, migration and invasion in NSCLC via absorbing miR-506-3p. Our findings provided a new biomarker for NSCLC therapy.

## Highlights

1. Knockdown of circ_100565 inhibits the progression of NSCLC;
2. Circ_100565 can sponge miR-506-3p;
3. MiR-506-3p can target HMGA2.

## Background

Lung cancer (LC) is a common primary lung malignancy with a high incidence of all malignancies [1]. Non-small cell lung cancer (NSCLC) is a common subtype of LC, accounting for about 80-85% of the total number of LC [2]. NSCLC begins to spread at an early stage, often metastasizes quickly and is prone to recurrence [3, 4]. At present, the prognosis of NSCLC patients is often poor, and the 5-year survival rate is less than 20% [5]. Therefore, finding new molecular targets for NSCLC may provide a new idea for the treatment of NSCLC.

Circular RNAs (circRNAs) are a type of non-coding RNAs with a covalently closed ring structure [6]. More and more studies have shown that circRNAs play a crucial function in the progression of various cancers, including NSCLC [7]. For example, hsa_circ_0023404 facilitated proliferation and metastasis in NSCLC [8], and circ_PRMT5 could promote the progression of NSCLC [9]. In our study, the microarray profiles of circRNAs identified that circ_100565 was elevated in NSCLC tissues compared with adjacent normal tissues. Therefore, circ_100565 was selected to explore its role in the progression of NSCLC.

Current studies have found that circRNAs play a role in a variety of mechanisms, among which the most proven one is as a sponge of microRNA (miRNA) to promote the expression of the downstream target gene [10, 11]. For instance, circ_0000218 could sponge miR-139-3p to promote RAB1A expression, thus regulating the proliferation and metastasis of colorectal cancer [12]. Also, circ_0005576 upregulated KIF20A to promote the progression of cervical cancer through sponging miR-153 [13]. In NSCLC, miR-217, miR-600 and miR-488-3p have also been shown to be involved in the regulation of circRNAs on NSCLC progression [8, 14, 15]. MiR-506-3p has been shown to be under-expressed in a variety of cancers, and can act as a tumor inhibitor to participate in the regulatory process of cancer, such as prostate cancer, retinoblastoma and osteosarcoma [16–18]. However, its function in NSCLC is still unclear. High mobility group AT-hook 2 (HMGA2) is a member of HMGA family, and previous studies have shown that HMGA overexpression is often found in malignant tumors, which is often associated with the transformation of tumor cells [19]. Therefore, HMGA2 often functions as an oncogene to involve in the regulation of cancer progression [20, 21].

In our study, we aimed to explore the function of circ_100565 in NSCLC, and determine its underlying mechanism, so as to provide new therapeutic targets or prognostic markers for NSCLC.

## Materials and methods

### Samples collection

NSCLC tissues (NSCLC) and adjacent normal tissues (Normal) were collected from 50 NSCLC patients who recruited from Huaihe Hospital of Henan University. All patients did not receive any treatment and signed informed consent. This study was authorized by the Ethics Committee of Huaihe Hospital of Henan University.

### Microarray analysis

Total RNAs were extracted from 3 pairs of NSCLC tissues and adjacent normal tissues by Trizol reagent (Invitrogen, Carlsbad, CA, USA). Then, messenger RNAs (mRNAs) were purified, amplified and transcribed into fluorescent complementary RNAs (cRNAs). Labeled-cRNAs were hybridized onto CapitalBio Technology Human CircRNA Array v2.0 (CapitalBio, Beijing, China). Then, Microarray Scanner (Agilent, Santa Clara, CA, USA) was used to identify the differentially expressed circRNAs.

### Quantitative real-time polymerase chain reaction (qRT-PCR)

After total RNAs were extracted, the complementary DNA (cDNA) was synthesized by cDNA Synthesis Kit (Vazyme, Nanjing, China). QRT-PCR was performed using SYBR Green (Invitrogen). The relative expression was calculated using 2^−ΔΔCt^ methods and normalized using glyceraldehyde 3-phosphate dehydrogenase (GAPDH) or U6. The primers were as follows: circ_100565, F 5′-CCACACAACCGTACCACCTAA-3′, R 5′-TATGCTGGCTGCTACTGGAG-3′; HMGA2, F 5′-GCGCCTCAGAAGAGAGGAC-3′, R 5′-GGTCTCTTAGGAGAGGGCTCA-3′; GAPDH, F 5′-AAGGTGAAGGTCGGAGTCAA-3′, R 5′-AATGAAGGGGTCATTGATGG-3′; miR-506-3p, F 5′-GCCACCACCATCAGCCATAC-3′, R 5′-GCACATTACTCTACTCAGAAGGG-3′; U6: F 5′-GCAGGAGGTCTTCACAGAGT-3′, R 5′-TCTAGAGGAGAAGCTGGGGT-3′.

### Cell culture

NSCLC cell lines (Calu-3, Calu-6, A549 and H1299) were purchased from American Type Culture Collection (ATCC, Manassas, VA, USA). The human normal bronchial epithelium cell line (HBE1) was bought from Crisprbio (Beijing, China). All cells were cultured in RPMI-1640 medium (Gibco, Carlsbad, CA, USA) containing 10% fetal bovine serum (FBS, Gibco) and 1% penicillin (100 U/mL)/streptomycin (100 μg/mL) at 37 °C with 5% CO_2_ incubator.

### Authenticity identification of circRNA

The extracted RNAs were incubated with Ribonuclease R (RNase R; Duma, Shanghai, China) for 20 min. Also, part of the RNA (not added RNase R) served as a blank control (mock). Then, the circ_100565 expression was detected by qRT-PCR analysis, and GAPDH was used as an endogenous control.

### Subcellular fractionation and localization

Cytoplasmic and Nuclear RNA Purification Kit (Amyjet, Wuhan, USA) was used to isolate and extract the cytoplasm and nuclear RNA of A549 and H1299 cells. Then, the expression of circ_100565 in the cytoplasm and nuclear of A549 and H1299 cells was measured by qRT-PCR. GAPDH and U6 served as the cytoplasm control and nuclear control, respectively.

### Cell transfection

Lentiviral short hairpin RNA (shRNA) targeting circ_100565 (sh-circ_100565#1/2), miRNA mimic or inhibitor, HMGA2 overexpression plasmid and their negative controls (con, miR-NC, anti-NC and vector) were obtained from Ribobio (Guangzhou, China). Lipofectamine 3000 (Invitrogen) was used to transfect all plasmids into A549 and H1299 cells.

### Cell proliferation assay

The proliferation of A549 and H1299 cells was determined using cell counting kit-8 (CCK-8) and colony formation assays. For CCK-8 assay, A549 and H1299 cells were plated into 96-well plates. At the indicated time, CCK-8 reagent (Genomeditech, Shanghai, China) was added to each well and incubated for 4 h. The absorbance at 450 nm was measured to evaluate the viability of A549 and H1299 cells. For colony formation assay, A549 and H1299 cells were plated into 6-well plates. After transfection, cells were incubated for 2 weeks. Then, A549 and H1299 cells were fixed with methanol and stained with crystal violet. The number of colonies (> 50 cells) was counted under a microscope (Shoif, Shanghai, China).

### Flow cytometry

This assay was used to measure the cell cycle distribution of cells. After transfection for 48 h, A549 and H1299 cells were harvested and collected into a centrifuge tube. Then, cells were fixed with 70% ethanol, incubated with RNase, and then stained with propidium iodide (PI; Beyotime, Shanghai, China). Finally, the cell cycle distribution was analyzed by a Flow Cytometer.

### Transwell assay

Transwell chambers with an 8-µm pore size (Corning Inc., Corning, NY, USA) was used to perform cell migration and invasion assays. The upper chambers non-coated Matrigel (BD Biosciences, San Jose, CA, USA) were used to detect migration, while pre-coated Matrigel were applied to detect invasion. Briefly, A549 and H1299 cells were plated into the upper chambers (without FBS), while the lower chambers were added medium (with FBS). After 24 h, cells were fixed and stained, and the number of migrated and invaded cells in lower chambers was counted under a microscope (Shoif).

### Western blot (WB) analysis

Total proteins were extracted by RIPA buffer (Beyotime). The proteins were subjected to sodium dodecyl sulfate–polyacrylamide gel electrophoresis (SDS-PAGE) gel and transferred onto polyvinylidene fluoride (PVDF) membranes (Membrane Solutions, Nantong, China). Then, the membranes were blocked with nonfat milk and incubated with primary antibodies against proliferating cell nuclear antigen (PCNA; 1:1000, Bioss, Beijing, China), matrix metalloproteinase 9 (MMP9; 1:500, Bioss), E-cadherin (1:2000, Bioss), Vimentin (1:1500, Bioss), HMGA2 (1:500, Bioss) and GAPDH (1:500, Bioss) overnight at 4 °C. After incubated with secondary antibody (1:1000, Bioss), the membranes were added with enhanced chemiluminescence solution (Beyotime) to visualize the protein blots.

### Mice xenograft models

Male BALB/c nude mice (6-week-old) were bought from Vital River (Beijing, China) and randomly divided into 2 experimental groups (n = 6 per/group). A549 cells were transfected with sh-circ_100565 or con and subcutaneously injected into nude mice. The tumor volume was calculated every 7 d using the formula: length × width^2^/2 method. After 35 d, the tumors were removed for further experiments. All animal procedures were approved by the Animal Committee of Huaihe Hospital of Henan University.

### Dual-luciferase reporter assay

The sequences of circ_100565 and HMGA2 3′UTR containing predicated miR-506-3p binding sites or mutant binding sites were synthesized by Generalbio (Anhui, China) to form the wild-type or mutant-type reporter vectors (circ_100565-WT and HMGA2-WT or circ_100565-MUT and HMGA2-MUT). The above reporter vectors were co-transfected with miR-506-3p mimic or miR-NC into A549 and H1299 cells using Lipofectamine 3000 (Invitrogen). After 48 h, the luciferase activities were tested using a Dual-luciferase Reporter Gene Assay (Beyotime).

### RNA immunoprecipitation (RIP) assay

A549 and H1299 cells were lysed using RIP lysis buffer (Millipore, Billerica, MA, USA). Then, cell lysate was incubated with magnetic beads (Millipore) conjugated with argonaute2 antibody (anti-ago2) or immunoglobulin G (IgG) antibody (anti-IgG). Part of cell lysates was not incubated with magnetic beads and served as a blank control (Input). The co-precipitated RNAs were purified and tested by qRT-PCR.

### Biotin-labeled pull-down assay

Biotin-labeled miR-506-3p (Bio-miR-506-3p) and negative control (Bio-miR-NC) were synthesized from Sangon Biotech (Shanghai, China). A549 and H1299 cells were transfected with the above probes and incubated for 48 h. After that, cells were lysed and incubated with magnetic beads at 4 °C for 3 h. Then, qRT-PCR was used to measure the enrichment of HMGA2 in Bio-miR-506-3p or Bio-miR-NC.

### Statistical analysis

All data were shown as the mean ± standard deviation. Student’s *t*-test or one-way analysis of variance was used for statistical analysis in SPSS17.0 software (SPSS Inc., Chicago, IL, USA). *P* < 0.05 was defined to be statistically significant.

## Results

### Circ_100565 was higher expressed in NSCLC tissues and cells

**Fig. 1:**
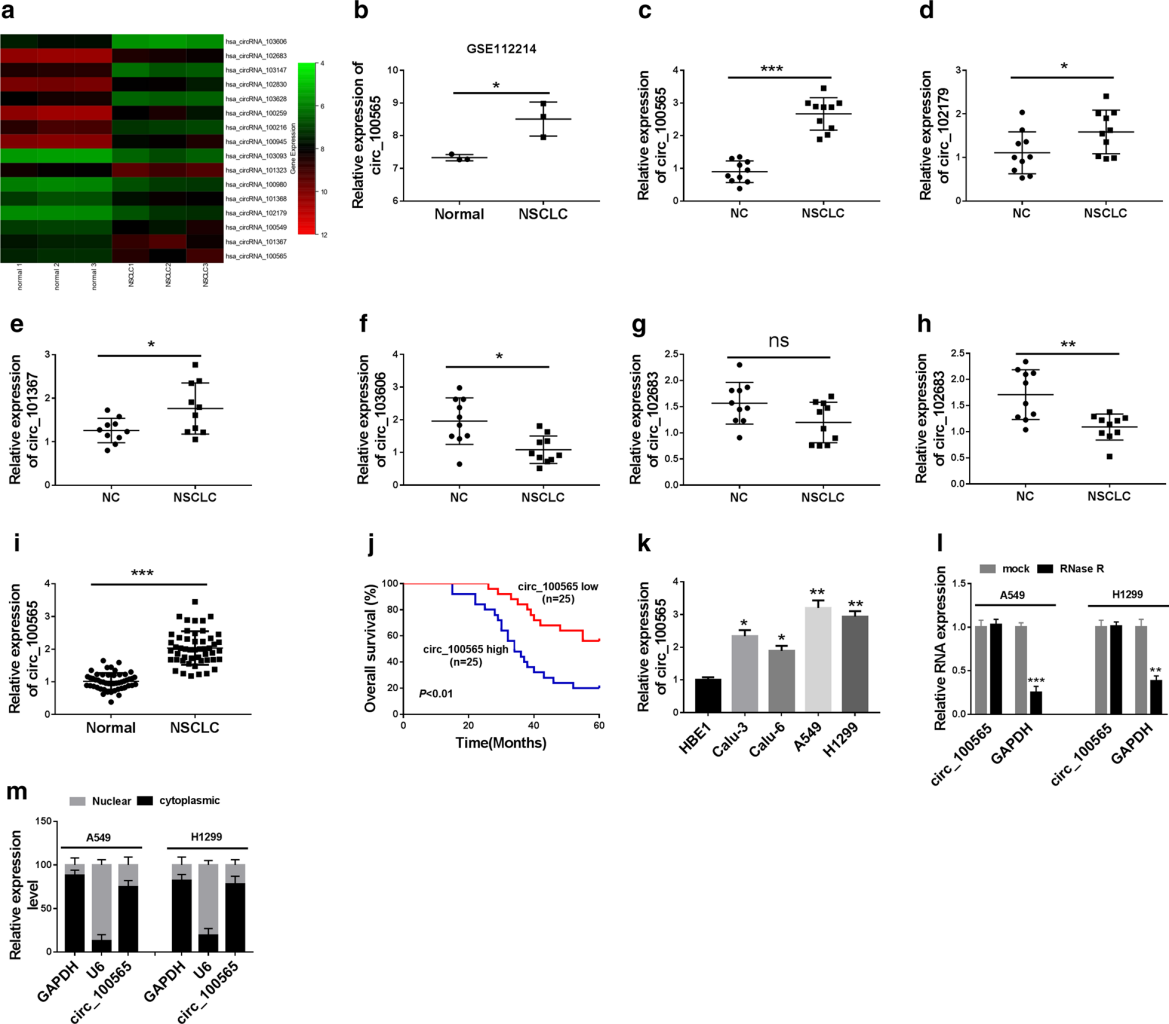
The expression of circ_100565 in NSCLC tissues and cells. **a** Heat map: differential expression of circRNAs in NSCLC tissues (NSCLC) and adjacent normal tissues (Normal). **b** Circ_100565 was markedly upregulated by more than 2.99-fold in NSCLC compared with that in Normal. **c**–**h** QRT-PCR was used to measure the first 3 up-regulated circRNAs and first 3 down-regulated circRNAs in 10 pairs of NSCLC tissues and normal tissues (NC). **i** The expression of circ_100565 in NSCLC and Normal was detected by qRT-PCR. **j** Kaplan–Meier analysis examined the correlation between circ_100565 expression and overall survival of NSCLC patients. **k** Circ_100565 expression in NSCLC cell lines (Calu-3, Calu-6, A549 and H1299) and HBE1 cells was determined by qRT-PCR. **l** The expression levels of circ_100565 and GAPD in A549 and H1299 cells were assessed by qRT-PCR to evaluate the authenticity of circ_100565. **m** Subcellular fractionation assay was used to measure the expression levels of circ_100565, U6 and GAPDH in the nuclear and cytoplasm of A549 and H1299 cells. *P < 0.05, **P < 0.01, ***P < 0.001

We used microarray analysis to identify the differentially expressed circRNAs in NSCLC tissues and adjacent normal tissues. According to the cut-off criteria (log2 |fold change| > 1 and *P* < 0.05), we found that there were 103 differentially expressed circRNAs, of which 8 were up-regulated and 95 were down-regulated in NSCLC. The 8 circRNAs that were most significantly up-regulated and the 8 circRNAs that were most significantly down-regulated in NSCLC tissues were taken for heat maps (Fig. 1a). Statistical analysis results showed that circ_100565 was 2.99 folds elevated in NSCLC tissues (Fig. 1b). Among the up- and down-regulated circRNAs, we selected the first 3 circRNAs to measure their expression levels in 10 pairs of NSCLC tissues. Among them, circ_100565 was most significantly up-regulated in NSCLC (Fig. 1c–h), so we chose circ_100565 for further research. In our results, we found that circ_100565 expression was markedly increased in NSCLC tissues compared with adjacent normal tissues (Fig. 1i). According to the median expression value of circ_100565 in NSCLC patients’ tissues, we divided the NSCLC tissues into high-expression group and low-expression group. The correlation between circ_100565 expression and the clinical pathological features of NSCLC patients suggested that high circ_100565 expression was positively correlated with the lymph node metastasis and TNM stages of NSCLC patients (*P *< 0.05, Table 1). Kaplan–Meier analysis revealed that high circ_100565 expression also was positively associated with poor overall survival of NSCLC patients (Fig. 1j). Moreover, we found that circ_100565 expression was higher in four NSCLC cell lines (especially A549 and H1299) than that in HBE1 cells (Fig. 1k). To further verify the circular property of circ_100565, we performed RNase R digestion assay in A549 and H1299 cells and the results showed that circ_100565 was resistant to the digestion of RNase R, whereas GAPDH mRNA was degraded by RNase R (Fig. 1l). In addition, we analyzed the subcellular distribution of circ_100565 and found that circ_100565 was mainly localized in the cytoplasm of NSCLC cells (Fig. 1m), which indicated that circ_100565 mainly played a regulatory role in the post-transcriptional level. All data revealed that circ_100565 might have an important function in NSCLC.

**Table 1:** Correlation between circ_100565 expression and the clinical pathological features of 50 NSCLC patients

Characteristic	All cases	circ_100565 expression		P-value
		High (n = 25)	Low (n = 25)	
Gender				0.569
Male	28	15	13	
Female	22	10	12	
Age (years)				0.684
< 60	7	3	4	
≥ 60	43	22	21	
Smoking status				0.774
Smokers	29	14	15	
No-smokers	21	11	10	
Differentiation				0.087
Well/moderate	28	17	11	
Poor	22	8	14	
Lymph node metastasis				0.011*
No	27	18	9	
Yes	23	7	16	
TNM stages				0.002*
I/II	23	17	6	
III/IV	27	8	19	

* *P *< 0.05

### Knockdown of circ_100565 inhibited proliferation, migration and invasion in NSCLC cells

**Fig. 2:**
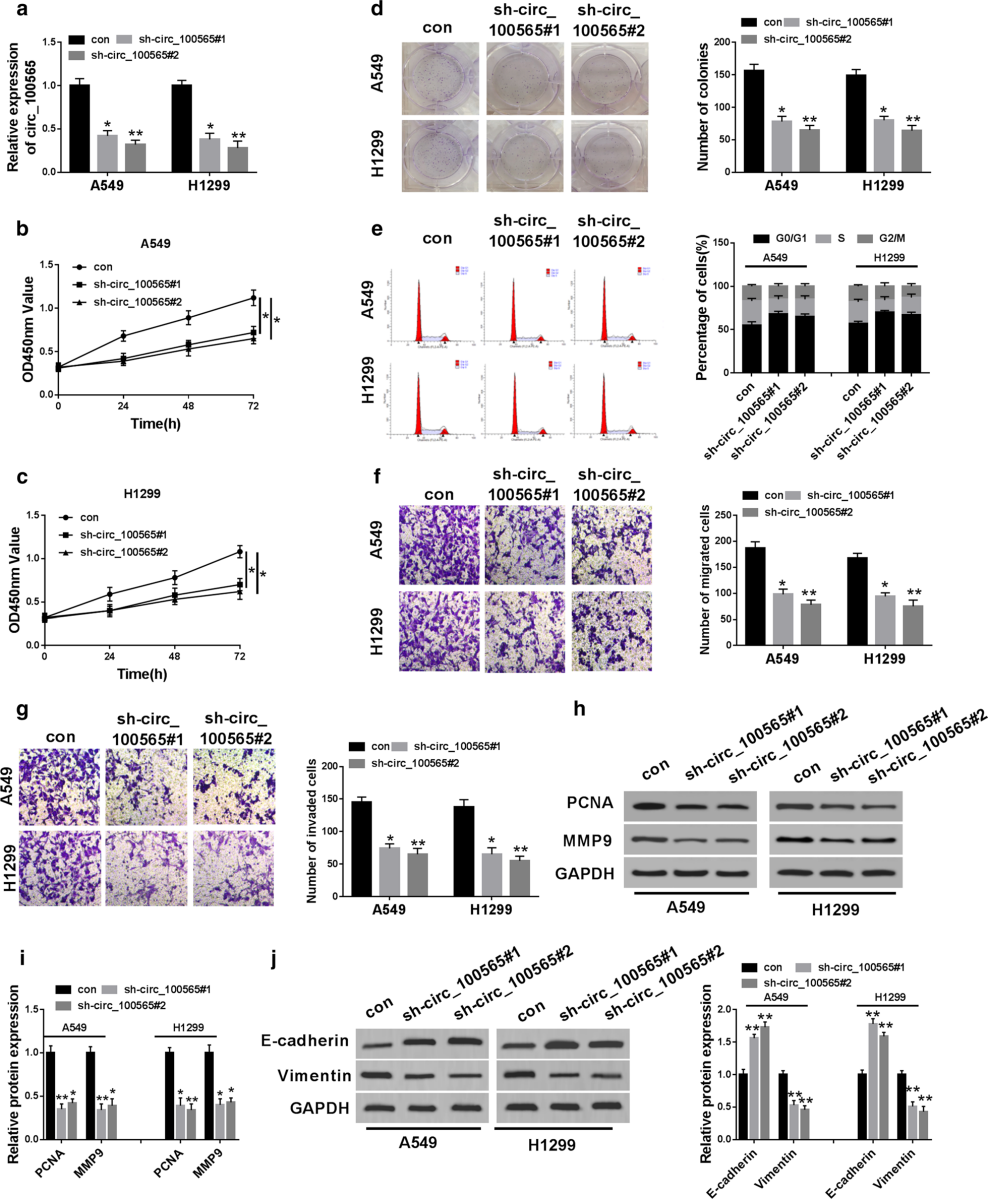
Effects of circ_100565 on the progression of NSCLC cells. A549 and H1299 cells were transfected with sh-circ_100565#1/2 or con. **a** The expression of circ_100565 was detected by qRT-PCR to evaluate transfection efficiency. **b**, **c** CCK-8 assay was performed to assess the viability of A549 and H1299 cells. **d** Colony formation assay was used to measure the number of colonies in A549 and H1299 cells. **e** The cell cycle distribution of A549 and H1299 cells was evaluated using flow cytometry. **f**, **g** The numbers of migrated and invaded A549 and H1299 cells were determined by transwell assay. **h**, **i** The protein levels of PCNA and MMP9 in A549 and H1299 cells were detected by WB analysis. **j** WB analysis was performed to measure the protein level of E-cadherin and Vimentin in A549 and H1299 cells. * *P* < 0.05, **P < 0.01

To explore the biological function of circ_100565 in NSCLC, we transfected sh-circ_100565#1/2 into A549 and H1299 cells, and the results showed that two shRNAs remarkably reduced the expression of circ_100565 in A549 and H1299 cells (Fig. 2a), indicating that the transfection was successful and the next functional experiments could be carried out. CCK-8 results showed that silenced circ_100565 markedly decreased the viability of A549 and H1299 cells (Fig. 2b, c), and colony formation assay revealed that the colonies of A549 and H1299 cells were also significantly reduced after circ_100565 silencing (Fig. 2d), indicating that circ_100565 knockdown significantly suppressed the proliferation of NSCLC cells. Moreover, through analyzing the cell cycle of A549 and H1299 cells, we confirmed that circ_100565 knockdown could induce cell cycle arrest in G0/G1 phase to reduce the percentage of cells in S phase (Fig. 2e). Furthermore, transwell assay results determined that the number of migrated and invaded A549 and H1299 cells was obviously repressed by circ_100565 depletion (Fig. 2f, g), suggesting that silenced circ_100565 restrained the migration and invasion of NSCLC cells. At the same time, the decreasing of proliferation marker protein PCNA and metastasis marker protein MMP9 levels further confirmed the inhibition of circ_100565 silencing on the proliferation, migration and invasion in A549 and H1299 cells (Fig. 2h, i). In addition, we also found that silenced circ_100565 could increase E-cadherin protein level and inhibit Vimentin protein level, showing that the EMT process of A549 and H1299 cells also could be suppressed by circ_100565 silencing (Fig. 2j). Therefore, these results revealed that circ_100565 might play an oncogenic role in NSCLC cells.

### Interference of circ_100565 reduced NSCLC tumor growth in vivo

**Fig. 3:**
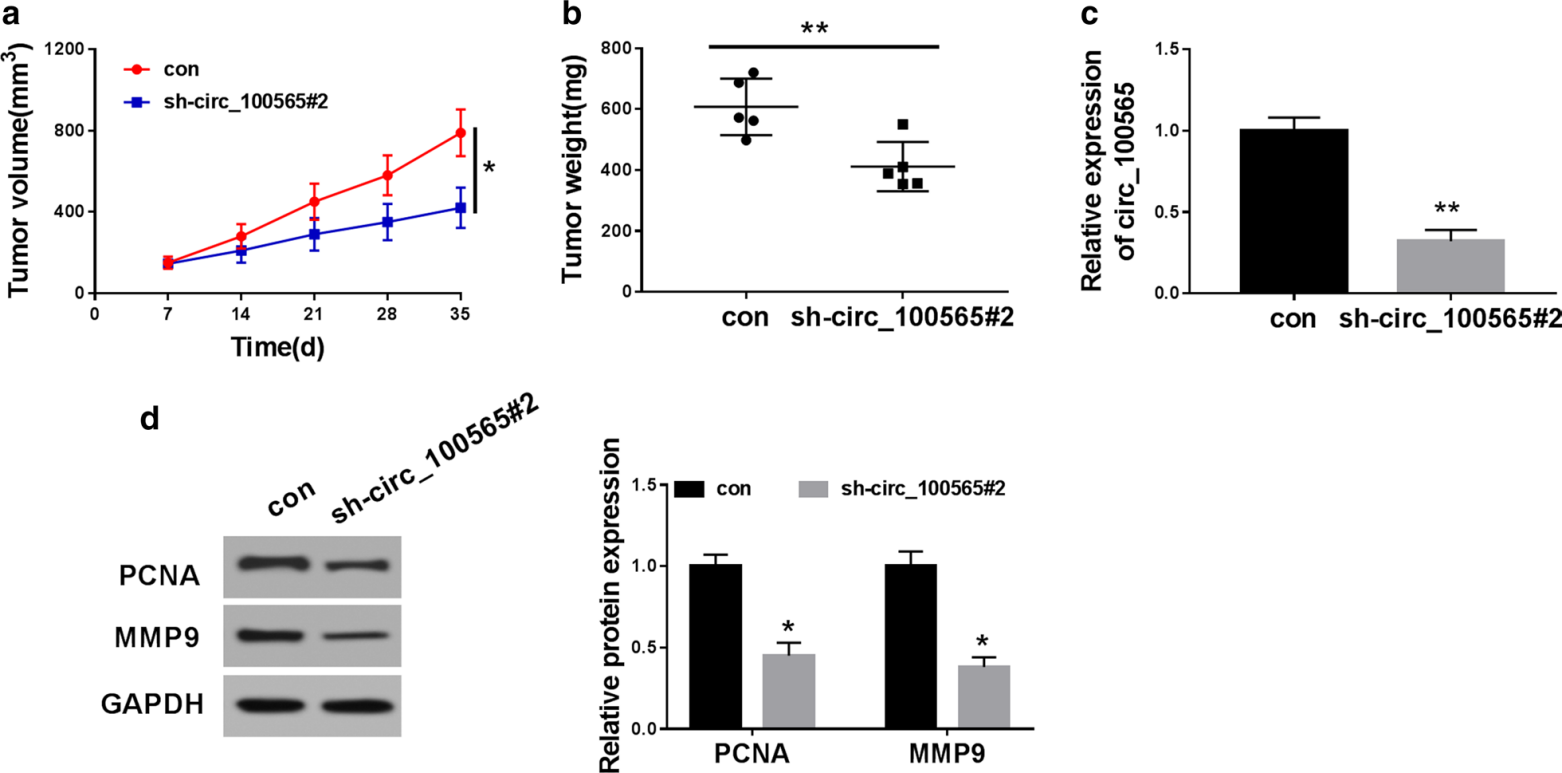
Effects of circ_100565 on NSCLC tumor growth in vivo. **a** Tumor volume was calculated with length × width^2^/2 method at the indicated time point. **b** Tumor weight was measured in mice. **c** The expression level of circ_100565 was detected by qRT-PCR to evaluate transfection efficiency. **d** The protein levels of PCNA and MMP9 were tested by WB analysis. * *P* < 0.05, **P < 0.01

To further confirm our conclusion, we performed in vivo experiments using the mice xenograft models. By measuring tumor volume, we found that the growth rate of tumor volume in the circ_100565 knockdown group was significantly inhibited compared with the control group (Fig. 3a). Besides, we also discovered that the tumor weight was markedly decreased in the circ_100565 knockdown group (Fig. 3b). QRT-PCR results showed that circ_100565 interference was successful in the sh-circ_100565#2 group (Fig. 3c). Moreover, we also detected the protein levels of PCNA and MMP9 in tumors and found that silenced circ_100565 obviously suppressed the protein levels of PCNA and MMP9 in tumors (Fig. 3d), indicating that circ_100565 inhibited the proliferation and metastasis of NSCLC tumor, thereby reducing the tumor growth of NSCLC. These data again confirmed that circ_100565 played an active function in NSCLC.

### Circ_100565 directly sponged miR-506-3p

**Fig. 4:**
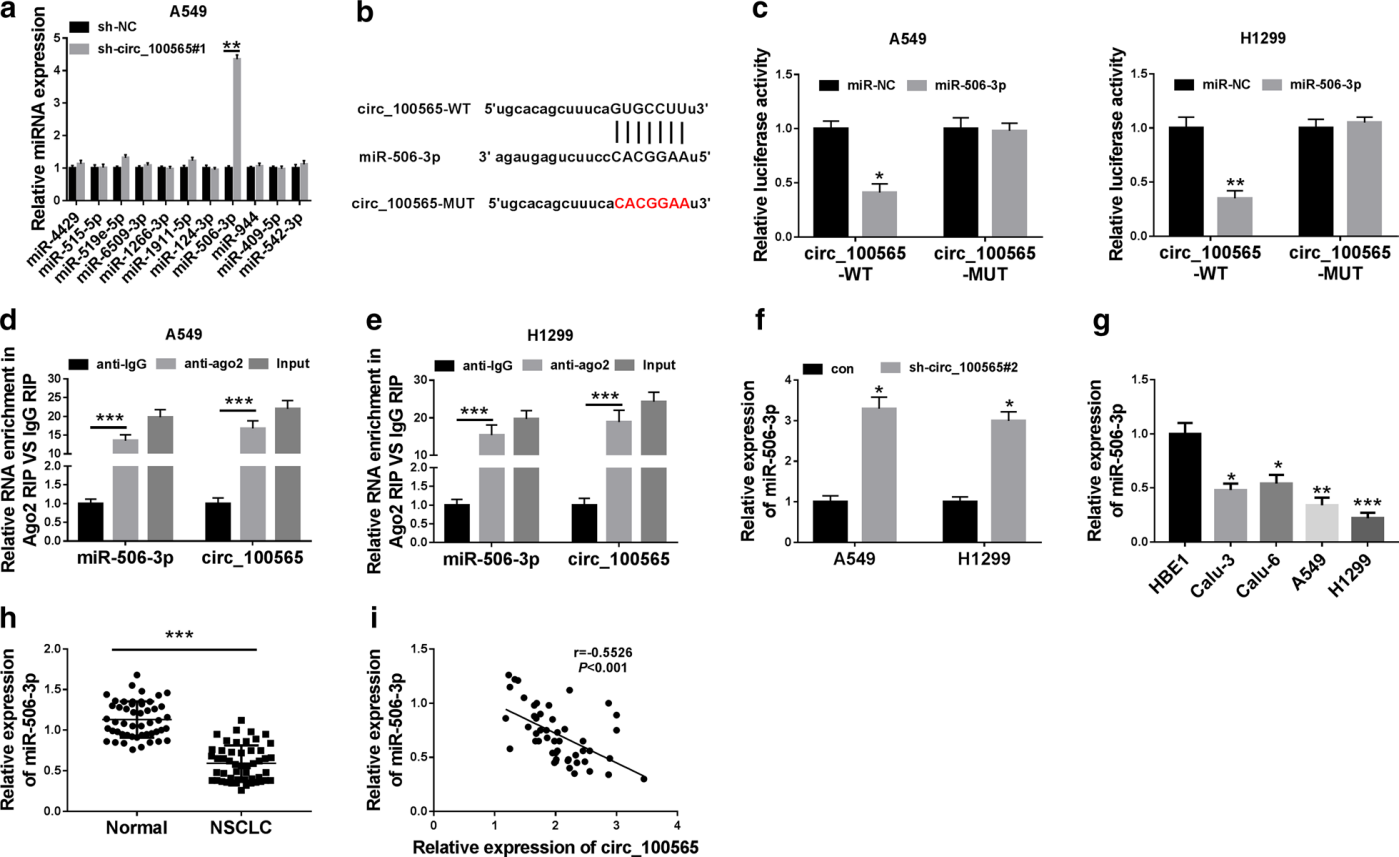
MiR-506-3p could be sponged by circ_100565. **a** The expression changes of 11 targeted miRNAs in A549 cells transfected with sh-circ_100565 or sh-NC were detected by qRT-PCR. **b** The sequences of circ_100565 contained the miR-506-3p binding sites or mutant binding sites were shown. **c**, **d** Dual-luciferase reporter assay was used to detect the interaction between miR-506-3p and circ_100565 in A549 and H1299 cells. **e** RIP assay was performed to determine the enrichment of circ_100565 and miR-506-3p in anti-ago2 or anti-IgG. **f** The effect of sh-circ_100565#2 on the expression of miR-506-3p in A549 and H1299 cells was detected by qRT-PCR. **g** The expression of miR-506-3p in NSCLC cell lines (Calu-3, Calu-6, A549 and H1299) and HBE1 cells was determined by qRT-PCR. **h** QRT-PCR was performed to assess the expression of circ_100565 in NSCLC tissues (NSCLC) and adjacent normal tissues (Normal). **i** The correlation between miR-506-3p and circ_100565 expression was measured by Pearson correlation analysis. *P < 0.05, **P < 0.01, ***P < 0.001

To perfect the function of circ_100565 as a miRNA sponge, we predicted the potential target miRNA of circ_100565 using the StarBase tool. The tool predicted that circ_100565 could target 11 miRNAs (miR-4429, miR-515-5p, miR-519e-5p, miR-6509-3p, miR-1266-3p, miR-1911-5p, miR-126-3p, miR-124-3p, miR-506-3p, miR-944, miR-409-5p and miR-542-3p). However, we detected the expression changes of targeted miRNAs after knocking down circ_100565, and found that miR-506-3p is most obviously upregulated (Fig. 4a), so miR-506-3p was selected for our research. The complementary binding sites between miR-506-3p and circ_100565 were shown in Fig. 4b. To validate the binding ability between circ_100565 and miR-506-3p, we performed the dual-luciferase reporter assay. The results demonstrated that miR-506-3p overexpression could significantly weaken the luciferase activity of circ_100565-WT in A549 and H1299 cells, but not circ_100565-MUT (Fig. 4c, d). Furthermore, RIP assay results also uncovered that in A549 and H1299 cells, circ_100565 and miR-506-3p were markedly enriched in anti-ago2 (Fig. 4e). Moreover, silenced circ_100565 markedly promoted the expression of miR-506-3p, suggesting that miR-506-3p expression was regulated by circ_100565 (Fig. 4f). Besides, we also detected the expression of miR-506-3p in NSCLC tissues and cells, and the results determined that miR-506-3p was lower expressed in NSCLC cells and tissues compared to the control (Fig. 4g, h). In addition, correlation analysis revealed that miR-506-3p expression was negatively correlated with circ_100565 in NSCLC tissues (Fig. 4i). Hence, the above results confirmed that miR-506-3p was absorbed by circ_100565 in NSCLC.

### MiR-506-3p could target HMGA2

**Fig. 5:**
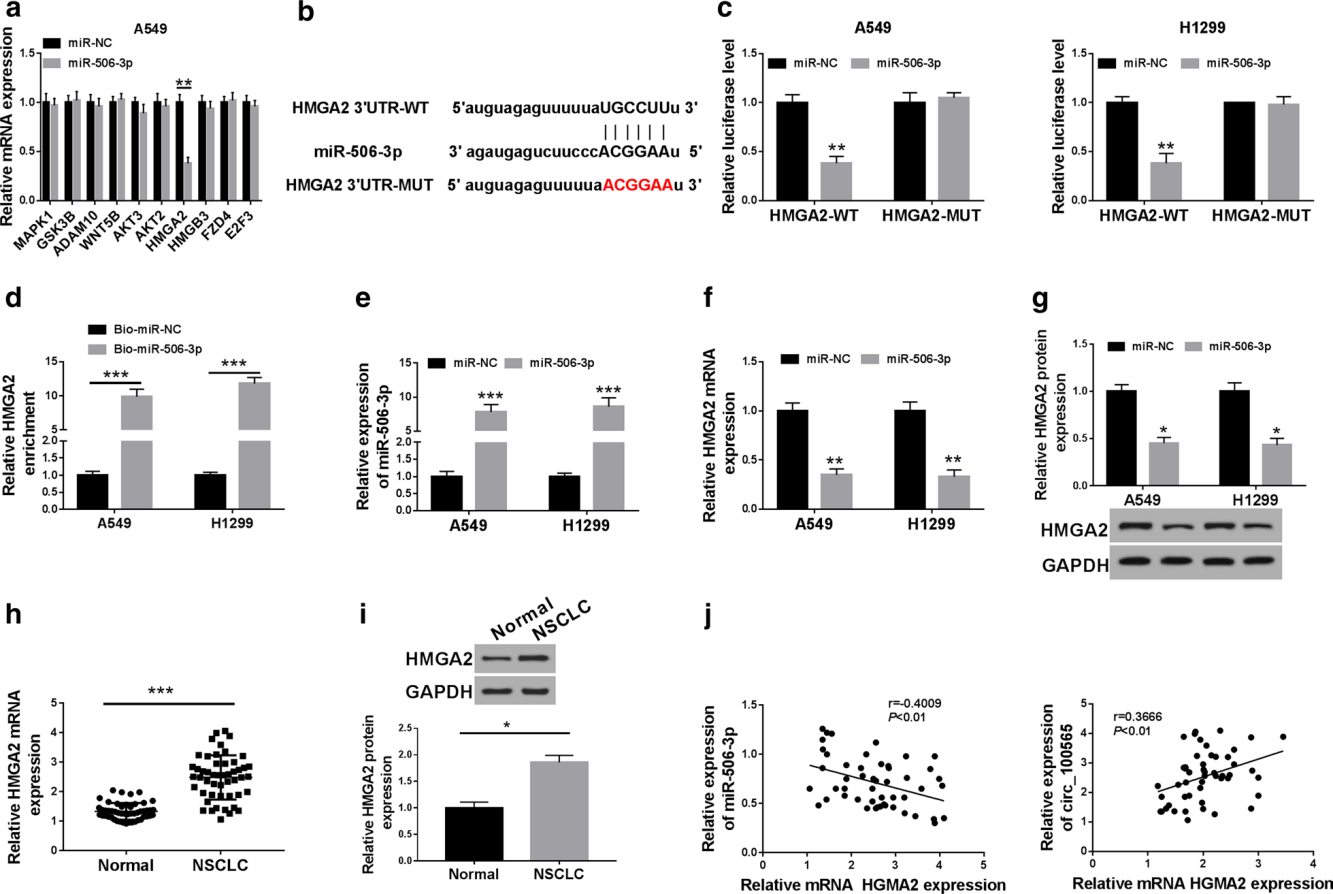
HMGA2 was a target of miR-506-3p. **a** The expression changes of 10 targeted genes in A549 cells transfected with smiR-506-3p or miR-NC were detected by qRT-PCR. **b** The sequences of miR-506-3p contained the HMGA2 3′UTR binding sites or mutant binding sites were shown. The interaction between miR-506-3p and HMGA2 in A549 or H1299 cells was assessed by dual-luciferase reporter assay (**c**) and biotin-labeled pull down (**d**). **e** The expression of miR-506-3p was detected by qRT-PCR to evaluate the transfection efficiency of miR-506-3p mimic. **f** The effect of miR-506-3p mimic on the mRNA expression of HMGA2 was measured by qRT-PCR in A549 and H1299 cells. **g** The effect of miR-506-3p mimic on the protein level of HMGA2 was detected by WB analysis in A549 and H1299 cells. **h**, **i** The mRNA and protein expression levels of HMGA2 in NSCLC tissues (NSCLC) and adjacent normal tissues (Normal) were determined by qRT-PCR and WB analysis. **j** The correlation between HMGA2 and miR-506-3p or circ_100565 expression was detected by Pearson correlation analysis. *P < 0.05, **P < 0.01, ***P < 0.001

At the same time, we also used the StarBase tool to predict the target genes of miR-506-3p. The tool predicted that miR-506-3p could target many genes. Through literature review, we selected 10 genes (MAPK1, GSK3B, ADAM10, WNT5B, AKT3, AKT2, HMGA2, HMGB3, FZD4 and E2F3) that played an important role in NSCLC for further verification. In our experiments, we found that overexpression of miR-506-3p significantly reduced the mRNA level of HMGA2 (Fig. 5a). Therefore, HMGA2 was selected for this study. The binding sites between HMGA2 3′UTR and miR-506-3p were presented in Fig. 5b. Dual-luciferase reporter assay results revealed that miR-506-3p overexpression remarkably suppressed the luciferase activity of HMGA2-WT in A549 cells, while had no effect on the luciferase activity of HMGA2-MUT (Fig. 5c). The results of biotin-labeled pull-down assay indicated that HMGA2 could be pulled down by Bio-miR-506-3p compared to Bio-miR-NC, confirming the interaction between HMGA2 and miR-506-5p in NSCLC cells (Fig. 5d). Moreover, we explored the effect of miR-506-3p expression on HMGA2 expression in A549 and H1299 cells. Through detecting the expression of miR-506-3p, we found that the transfection efficiency of miR-506-3p mimic was better (Fig. 5e). QRT-PCR and WB analysis results showed that miR-506-3p overexpression obviously suppressed the mRNA and protein expression of HMGA2 in A549 and H1299 cells (Fig. 5f, g). Also, we detected the expression of HMGA2 in NSCLC tissues and adjacent normal tissues and discovered that the mRNA and protein expression levels of HMGA2 were upregulated in NSCLC tissues (Fig. 5h, i). In addition, correlation analysis indicated that HMGA2 expression was negatively correlated with miR-506-3p and positively correlated with circ_100565 in NSCLC tissues (Fig. 5j). Therefore, these results suggested that HMGA2 was a target of miR-506-3p in NSCLC.

### Circ_100565 regulated the progression of NSCLC cells through the miR-506-3p/HMGA2 axis

**Fig. 6:**
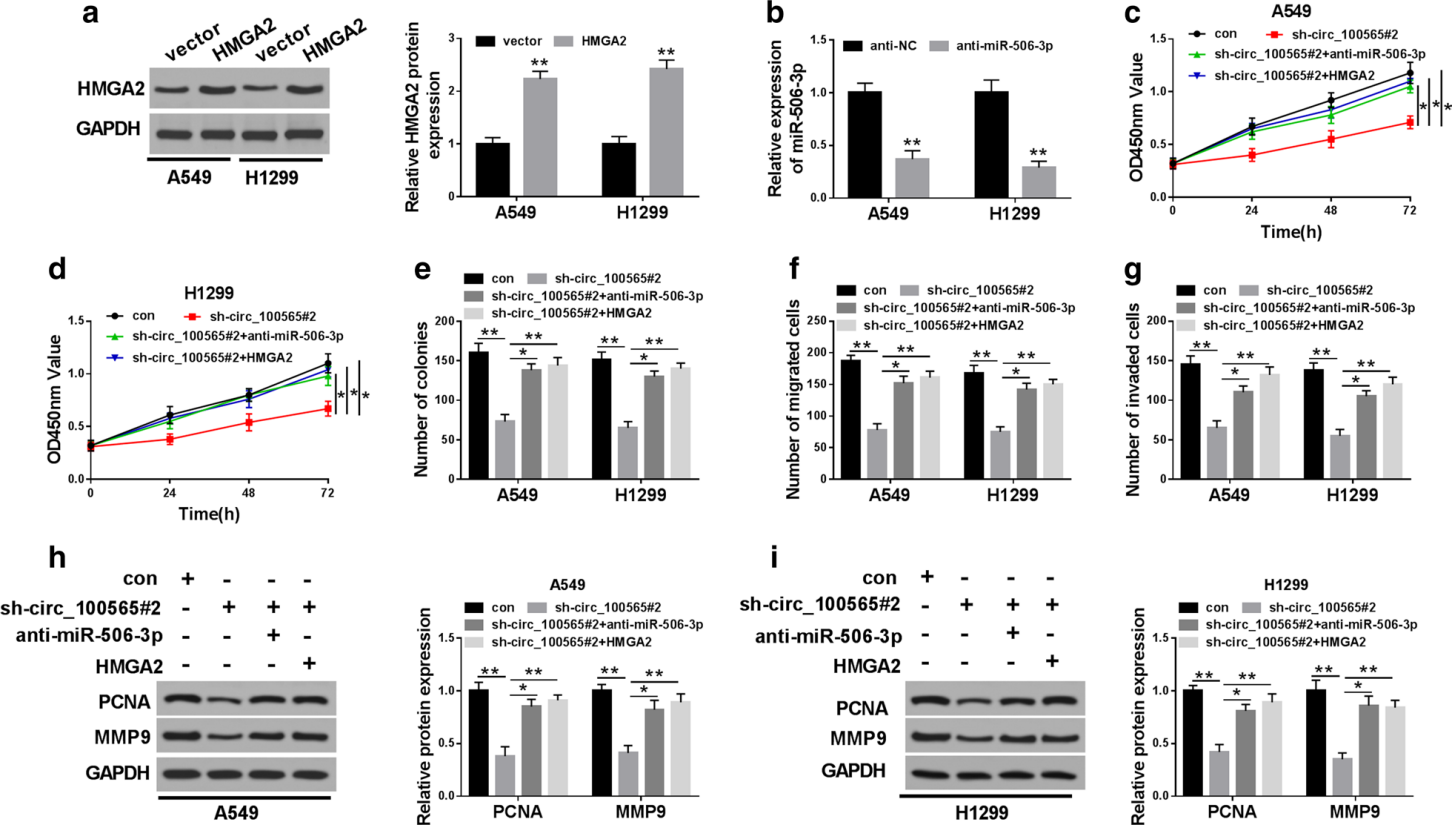
Effects of miR-506-3p and HMGA2 on the progression of NSCLC cells. A549 and H1299 cells were transfected with sh-circ_100565#2 and anti-miR-506-3p or HMGA2 overexpression plasmid. **a** The protein level of HMGA2 was measured by WB analysis to evaluate the transfection efficiency of HMGA2 overexpression plasmid. **b** The expression of miR-506-3p was detected by qRT-PCR to evaluate the transfection efficiency of anti-miR-506-3p. **c**, **d** The viability of A549 and H1299 cells was determined by CCK-8 assay. **e** Colony formation assay was performed to detect the number of colonies in A549 and H1299 cells. **f**, **g** Transwell assay was used to assess the numbers of migrated and invaded A549 and H1299 cells. **h**, **i** WB analysis was carried out to measure the protein levels of PCNA and MMP9 in A549 and H1299 cells. * *P* < 0.05, **P < 0.01

For further testing, we first detected the transfection efficiency of HMGA2 overexpression plasmid and anti-miR-506-3p through qRT-PCR and WB analysis, and found that HMGA2 overexpression plasmid had a better promotion effect on HMGA2 expression (Fig. 6a), and anti-miR-506-3p had a good inhibition effect on miR-506-3p expression in A549 and H1299 cells (Fig. 6b), indicating that the transfection of both was successful. To further verify that the function of circ_100565 on NSCLC through miR-506-3p and HMGA2, we co-transfected sh-circ_100565#2 and anti-miR-506-3p or HMGA2 overexpression plasmid into A549 and H1299 cells. CCK-8 and colony formation assays results revealed that the viability and colonies of A549 and H1299 cells were promoted in the sh-circ_100565 and anti-miR-506-3p or HMGA2 overexpression plasmid co-transfected groups compared with the sh-circ_100565 group, suggesting that miR-506-3p silencing or HMGA2 overexpression reversed the suppression effect of circ_100565 knockdown on proliferation in A549 and H1299 cells (Fig. 6c–e). Besides, transwell assay results determined that the addition of anti-miR-506-3p or HMGA2 overexpression plasmid also enhanced the number of migrated and invaded cells compared to the sh-circ_100565 group, showing that silenced-miR-506-3p or overexpressed-HMGA2 could invert the inhibition effects of circ_100565 knockdown on migration and invasion in A549 and H1299 cells (Fig. 6f, g). Furthermore, the promotion effects of anti-miR-506-3p or HMGA2 on the protein levels of PCNA and MMP9 also confirmed that miR-506-3p inhibition or HMGA2 overexpression could reverse the inhibition function of circ_100565 silencing on proliferation and metastasis in NSCLC cells (Fig. 6h, i). All results demonstrated that the miR-506-3p/HMGA2 axis was crucial to maintaining the function of circ_100565 in NSCLC cells.

## Discussion

At present, the pathogenic factors of NSCLC are not completely clear, but there are many risk factors, including smoking and occupational diseases [22]. Cancer development is accompanied by many gene interactions, including circRNA regulation [23]. The development of bioinformatics has contributed to the discovery of new circRNAs [24, 25]. In this study, we uncovered the role of circ_100565 in NSCLC. Our study revealed that and high expression of circ_100565 was closely related to the poor overall survival of NSCLC patients. Circ_100565 knockdown reduced the proliferation and metastasis of NSCLC cells in vitro and tumor growth in vivo. Further studies had shown that circ_100565 could absorb miR-506-3p to promote oncogene HMGA2 expression, thus promoting NSCLC progression. Hence, our studies revealed the importance of circ_100565 in the progression of NSCLC.

Although the roles of many circRNAs have been demonstrated in cancer, the importance of new circRNA discoveries cannot be ignored [26]. This study is the first to confirm the effect of circ_100565 on the proliferation, migration and invasion of NSCLC cells and verify the function of it on NSCLC tumor growth, which identifies the important role of circRNAs in cancer and adds a new member for the development of circRNAs.

MiRNAs mainly bind to target genes to regulate gene expression, and circRNA functions as a miRNA sponge have been well documented [27, 28]. To further perfect the mechanism of circ_100565, we predicted the adsorbed miRNA and found that miR-506-3p could be sponged by circ_100565, as demonstrated by dual-luciferase reporter and RIP assays. The function of miR-506-3p had been demonstrated in many cancers, and we discovered that it was also lower expressed in NSCLC, which was consistent with its expression in other cancers [16–18]. Besides, HMGA2, a well-known oncogene, could be targeted by miR-506-3p. The function of HMGA2 in the progression of NSCLC was also verified by Dai et al. and Li et al. [29, 30]. HMGA2 expression was correlated with miR-506-3p and circ_100565, implying that the circ_100565/miR-506-3p/HMGA2 axis was existed in NSCLC. This idea was also confirmed by further experiments that miR-506-3p inhibitor or HMGA2 overexpression obviously reversed the inhibition effects of circ_100565 knockdown on the proliferation, migration and invasion of NSCLC cells. The elucidation of the circ_100565 mechanism also provided references for studying the role of circ_100565 in other cancers.

Of course, the current research results also have some limitations. Our verification of the circ_100565/miR-506-3p/HMGA2 axis only remained at the cell level, and whether miR-506-3p inhibitor or HMGA2 overexpression can also invert the regulation of circ_100565 on NSCLC tumor growth in vivo is still unknown. Therefore, future experiments will further verify the circ_100565/miR-506-3p/HMGA2 axis in vivo.

## Conclusion

In conclusion, our results suggested that circ_100565 might function as an oncogene to promote NSCLC progression by regulating the miR-506-3p/HMGA2 axis. The discovery of circ_100565 might provide a new approach for targeted therapy of NSCLC.

## Data Availability

All data generated or analysed during this study are included in this published article.

## Abbreviations

circRNAs: Circular RNAs
NSCLC: Non-small cell lung cancer
miR-506-3p: microRNA-506-3p
HMGA2: High mobility group AT-hook 2
qRT-PCR: Quantitative real-time polymerase chain reaction
WB: Western blot
LC: Lung cancer
circRNAs: Circular RNAs
miRNA: microRNA

## References

[1] Bray F, Ferlay J, Soerjomataram I, Siegel RL, Torre LA, Jemal A (2018). Global cancer statistics 2018: GLOBOCAN estimates of incidence and mortality worldwide for 36 cancers in 185 countries. Cancer J Clin..

[2] Postmus PE, Kerr KM, Oudkerk M, Senan S, Waller DA, Vansteenkiste J, Escriu C, Peters S, Committee EG (2017). Early and locally advanced non-small-cell lung cancer (NSCLC): ESMO Clinical Practice Guidelines for diagnosis, treatment and follow-up. Annal Oncol..

[3] Herbst RS, Morgensztern D, Boshoff C (2018). The biology and management of non-small cell lung cancer. Nature.

[4] Ettinger DS, Akerley W, Borghaei H, Chang AC, Cheney RT, Chirieac LR, D’Amico TA, Demmy TL, Govindan R, Grannis FW (2013). Non-small cell lung cancer, version 2.2013. JNCCN..

[5] Ma PC (2012). Personalized targeted therapy in advanced non-small cell lung cancer. Clevel Clin J Med.

[6] Li X, Yang L, Chen LL (2018). The biogenesis, functions, and challenges of circular RNAs. Mol Cell.

[7] Ng WL, Mohd Mohidin TB, Shukla K (2018). Functional role of circular RNAs in cancer development and progression. RNA Biol.

[8] Liu C, Zhang Z, Qi D (2019). Circular RNA hsa_circ_0023404 promotes proliferation, migration and invasion in non-small cell lung cancer by regulating miR-217/ZEB1 axis. OncoTargets and therapy..

[9] Wang Y, Li Y, He H, Wang F (2019). Circular RNA circ-PRMT5 facilitates non-small cell lung cancer proliferation through upregulating EZH2 via sponging miR-377/382/498. Gene.

[10] Shang Q, Yang Z, Jia R, Ge S (2019). The novel roles of circRNAs in human cancer. Mol Cancer..

[11] Tay Y, Rinn J, Pandolfi PP (2014). The multilayered complexity of ceRNA crosstalk and competition. Nature.

[12] Pei FL, Cao MZ, Li YF (2019). Circ_0000218 plays a carcinogenic role in colorectal cancer progression by regulating miR-139-3p/RAB1A axis. J Biochem.

[13] Ma H, Tian T, Liu X, Xia M, Chen C, Mai L, Xie S, Yu L (2019). Upregulated circ_0005576 facilitates cervical cancer progression via the miR-153/KIF20A axis. Biomed Pharmacother.

[14] Chi Y, Luo Q, Song Y, Yang F, Wang Y, Jin M, Zhang D (2019). Circular RNA circPIP5K1A promotes non-small cell lung cancer proliferation and metastasis through miR-600/HIF-1alpha regulation. J Cell Biochem.

[15] Wan J, Hao L, Zheng X, Li Z (2019). Circular RNA circ_0020123 promotes non-small cell lung cancer progression by acting as a ceRNA for miR-488-3p to regulate ADAM9 expression. Biochem Biophys Res Commun.

[16] Hu CY, You P, Zhang J, Zhang H, Jiang N (2019). MiR-506-3p acts as a novel tumor suppressor in prostate cancer through targeting GALNT4. Eur Rev Med Pharmacol Sci.

[17] Han N, Zuo L, Chen H, Zhang C, He P, Yan H (2019). Long non-coding RNA homeobox A11 antisense RNA (HOXA11-AS) promotes retinoblastoma progression via sponging miR-506-3p. OncoTargets Ther.

[18] Wang D, Bao F, Teng Y, Li Q, Li J (2019). MicroRNA-506-3p initiates mesenchymal-to-epithelial transition and suppresses autophagy in osteosarcoma cells by directly targeting SPHK1. Biosci Biotechnol Biochem.

[19] De Martino M, Fusco A, Esposito F (2019). HMGA and cancer: a review on patent literatures. Recent Pat Anti-Cancer Drug Discov.

[20] Guo Z, He C, Yang F, Qin L, Lu X, Wu J (2019). Long non-coding RNA-NEAT1, a sponge for miR-98-5p, promotes expression of oncogene HMGA2 in prostate cancer. Biosci Rep.

[21] Tong H, Zhuang X, Cai J, Ding Y, Si Y, Zhang H, Shen M (2019). Long noncoding RNA ZFAS1 promotes progression of papillary thyroid carcinoma by sponging miR-590-3p and upregulating HMGA2 expression. OncoTargets Ther.

[22] Kang JK, Seo S, Jin YW (2019). Health effects of radon exposure. Yonsei Med J.

[23] Zhang HD, Jiang LH, Sun DW, Hou JC, Ji ZL (2018). CircRNA: a novel type of biomarker for cancer. Breast cancer..

[24] Zhang S, Zeng X, Ding T, Guo L, Li Y, Ou S, Yuan H (2018). Microarray profile of circular RNAs identifies hsa_circ_0014130 as a new circular RNA biomarker in non-small cell lung cancer. Sci Rep.

[25] Jiang MM, Mai ZT, Wan SZ, Chi YM, Zhang X, Sun BH, Di QG (2018). Microarray profiles reveal that circular RNA hsa_circ_0007385 functions as an oncogene in non-small cell lung cancer tumorigenesis. J Cancer Res Clin Oncol.

[26] Bach DH, Lee SK, Sood AK (2019). Circular RNAs in Cancer. Molecular therapy Nucleic acids..

[27] Verduci L, Strano S, Yarden Y, Blandino G (2019). The circRNA-microRNA code: emerging implications for cancer diagnosis and treatment. Mol Oncol.

[28] Arnaiz E, Sole C, Manterola L, Iparraguirre L, Otaegui D, Lawrie CH (2019). CircRNAs and cancer: biomarkers and master regulators. Semin Cancer Biol.

[29] Dai FQ, Li CR, Fan XQ, Tan L, Wang RT, Jin H (2019). miR-150-5p inhibits non-small-cell lung cancer metastasis and recurrence by targeting HMGA2 and beta-catenin signaling. Mol Ther Nucleic Acids.

[30] Li XX, Di X, Cong S, Wang Y, Wang K (2018). The role of let-7 and HMGA2 in the occurrence and development of lung cancer: a systematic review and meta-analysis. Eur Rev Med Pharmacol Sci.

